# Pernicious Effects of Toe Sucking Habit in Children

**DOI:** 10.1155/2016/2475784

**Published:** 2016-11-29

**Authors:** Deepika Pai, Saurabh Kumar, Abhay T. Kamath, Vipin Bhaskar

**Affiliations:** ^1^Department of Pedodontics & Preventive Dentistry, Manipal College of Dental Sciences, Manipal, Manipal University, Manipal, India; ^2^Department of Oral and Maxillofacial Surgery, Manipal College of Dental Sciences, Manipal, Manipal University, Manipal, India; ^3^Department of Pedodontics & Preventive Dentistry, Mahe Institute of Dental Sciences, Mahe, Kerala, India

## Abstract

Digit sucking is common nonnutritive sucking habit in childhood. However it is unusual to find toe sucking habit in children. We report a case of a seven-year-old child sucking great toe of the left foot. The child was referred by her paediatrician for dental evaluation due to her complaint of recurrent episodes of pyrexia. A dental evaluation was warranted as no particular system contributed to such recurrent episodes of fever in this child. Although dental examination did not reveal any cause for recurrent episodes of pyrexia, as a part of routine history taking we discovered that this child indulges frequently in sucking the great toe of her left foot since infancy. Any nonnutritive sucking habit is considered deleterious; this habit also caused significant effect on the child's dentofacial structures, sucked toe, and her general health. Hence the treatment plan was formulated for immediate cessation of habit. Appropriate interception of habit and timely orthodontic intervention led to not only early interception of cross-bite but also decrease in pyrexial episodes. This case report describes the pernicious effects of toe sucking habit and its relevance to recurrent pyrexia in children.

## 1. Introduction

Digit sucking habit is characterized by the placement of one or more digits to varying depths in the mouth. This habit develops in uterine life and continuation of habit after age of four-five years can cause deleterious effects on dentofacial structures and sucked digit [1, 2]. The reported incidence of digit sucking habit ranges from 13 to 100% in infancy and up to 61–90% in early childhood [3, 4].

Several unusual cases of nonnutritive sucking habits have been reported in literature. Inclusive of such reports are sucking of forearm resulting in dentofacial deformities and keloid formation of sucked area [5]. A case report on digit sucking habit in a child with cleft lip and palate reported splaying of nasal and alveolar segments of clefts under the influence of aberrant pressure created by sucking habit which significantly affected their treatment planning for rehabilitation of cleft lip and palate [6]. Some authors have reported development of onychophagia resulting from transference of earlier existing thumb sucking habit [7]. It was also found that prolonged intense parafunctional habit like digit sucking habit caused gingival recession and pathologic migration of teeth [8]. Also, there have been reports of correlation of chronic parasitic infections due to prevalence of digit sucking habit affecting the general wellbeing of the children [9]. These unusual case reports have widen the arena of identifying unusual parafunctional habit as etiological factors for unusual or at times common dental problems and associated with general health of a child. We hereby report one such unusual case of pernicious effects of toe sucking habit and its relevance to recurrent pyrexia.

## 2. Case Report

A seven-year-old girl was referred by her paediatrician with complaint of recurrent pyrexia of untraceable origin. Since low grade chronic dental infections can cause repeated episodes of fever, the paediatrician desired for a dental evaluation. During history taking the patient's mother revealed recurrent episodes of fever ranging from 101 to 102 degrees Fahrenheit with two episodes in a month for the past one year. The fever subsided on taking medication. Adding further she also gave a history of occasional abdominal pain. Upon systemic review with her paediatrician we learnt that she had no history of shortness of breath, chest pain, diarrhoea, rashes, sore throat, ear ache, or urinary symptoms that could indicate the system responsible for such recurrent episodes of pyrexia. The paediatrician also revealed that her chest X-ray and blood profile were normal. There were no abnormalities detected in her urine and stool analysis as well.

**Figure 1:**
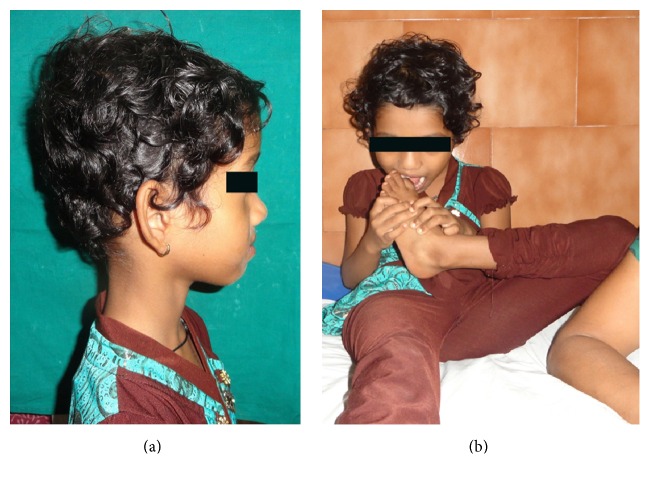
(a) Picture showing convex facial profile. (b) Picture showing child sucking the great toe of the left feet.

**Figure 2:**
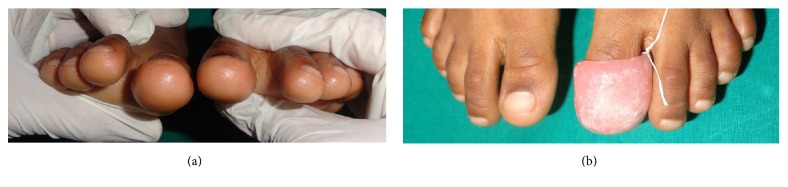
(a) Picture showing deformed great toe of left feet. (b) Picture showing acrylic toe guard fitted onto the great toe of left feet.

On examination her facial profile was convex (Figure 1(a)) with normal overjet and overbite. Intraoral examination did not reveal any deep carious lesions or any soft tissue abnormalities or palpable lymph nodes that could have been responsible for recurrent episodes of fever. On taking history for oral habits, the mother stated that the child sucks the great toe of her left feet since infancy (Figure 1(b)). On examination, the great toe of her left foot was deformed compared to that of the right foot (Figure 2(a)) which led to the diagnosis of toe sucking habit and its possible implication on recurrent episodes of fever. Since the mother and child were supportive to drop the habit, the detailed treatment plan was formulated.

On the next visit the parent and the patient were counselled for stopping the habit and they were educated on how it could be deleterious for the developing orofacial structures and on general health of the child. As a reminder therapy, the child was instructed to cover her foot with socks. In the subsequent visit upon finding the patient was reluctant to wear the socks, we implemented the method of negative reinforcement and reminder therapy which was accomplished with a toe guard fabricated with acrylic which fitted the great toe involved in sucking habit (Figure 2(b)).

**Figure 3:**
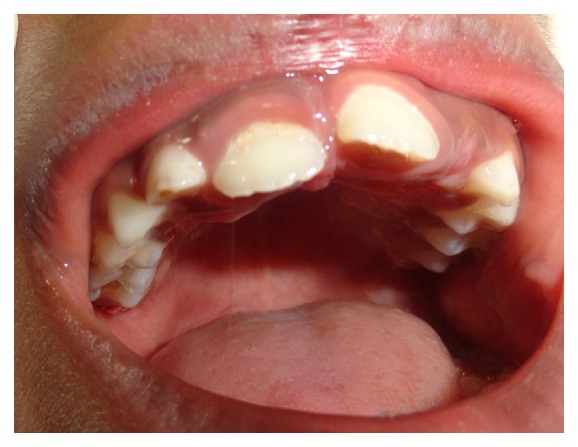
Picture showing right central incisor erupting in an abnormal pathway.

**Figure 4:**
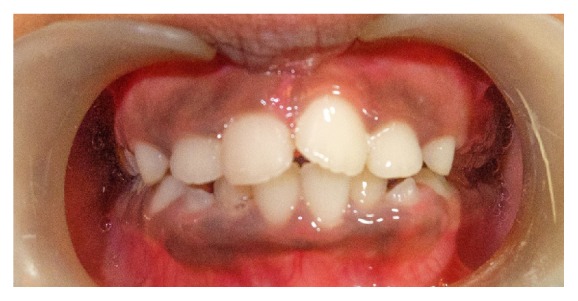
Picture showing right central incisor not in cross-bite and distoangular rotation of upper central incisors.

**Figure 5:**
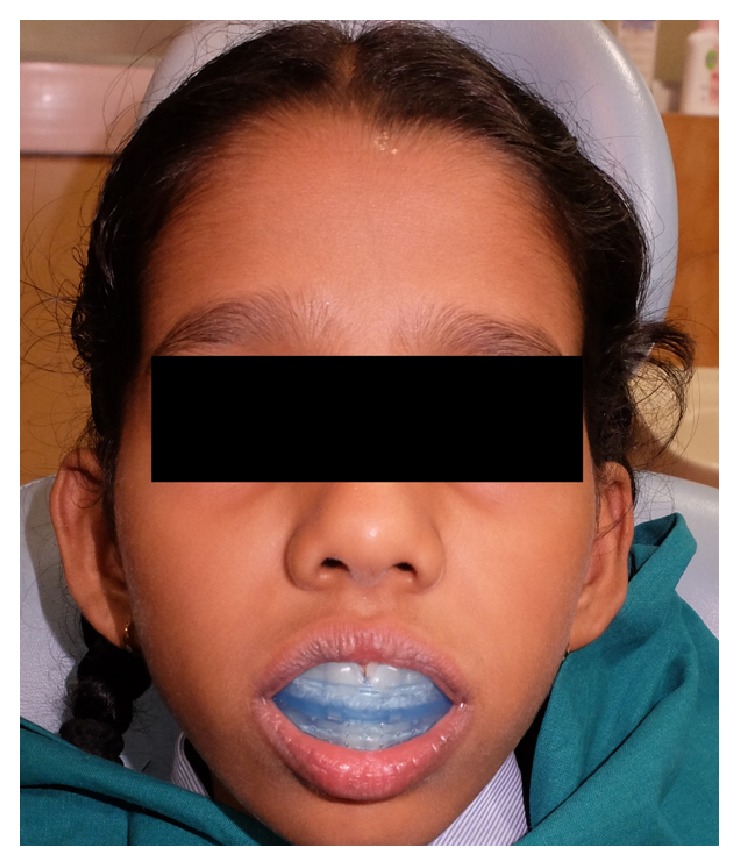
Picture showing patient wearing preorthodontic trainer for correction of distoangular rotation.

By three weeks the patient reported reduced frequency of toe sucking habit and episodes of fever. By now the upper incisor showed abnormal pathway of eruption (Figure 3) probably due to abnormal pressure exerted by the toe during sucking event that led to deflection of the developing tooth to an abnormal path. This developing cross-bite was intervened by tongue blade therapy. In the subsequent follow-up visit we noticed that the upper incisors showed distoangular rotation as they erupted into occlusion (Figure 4). The patient is currently using a preorthodontic trainer (T4K® Phase I, Preorthodontic Trainer for Kids, MRC, Australia) (Figure 5). The preorthodontic trainer was advocated to realign the incisors as well for myofunctional retraining of the oral musculature.

As of now the mother and the child did not report any secondary or substitutional habit after the cessation of toe sucking habit. Also no episodes of fever have been reported.

## 3. Discussion

The sucking habit develops as a result of rooting reflex in a neonate, which is an inherent biologic drive for sucking. The development of hand to mouth movements is described as a result of complex neurological development as well as muscular development stimulated by proprioceptive and rooting reflexes [10]. In early fetal life fore and hind limbs do not have distinguished functions; therefore it is not uncommon to find infants sucking the toe. As in our case also, the mother gave the history of toe sucking since infancy. It is when the child learns to stand and walk; the risk brought about by sucking of hand or toe may vary. Along with its deleterious effects on dentofacial structures, systemic wellbeing of the child can also be affected by the toe sucking habit [9].

Recurrent pyrexia can usually be triggered by environmental causes, with definite source of exposure. It is commonly seen in preschooler children attending day care centres [11, 12]. Since the children are exposed to outdoor activities the digit and the toe might act as a carrier for different microorganisms which may affect the general health of the patient.

In our case no definitive diagnosis could have been established for the recurrent episodes of fever since investigations like blood culture, chest X-ray, and USG abdomen were not conclusive.

According to few reported cases, allergic rhinitis and parasitic infections are attributed to be caused by digit sucking habit [9]. Analogously we can hypothesise that toe sucking habit as in our case could have led to recurrent pyrexia. The sucking of toe leads to contaminated source through establishment of a possible oropedal route leading to a wide range of parasitic, bacterial, and other agents to cause involvement of different systems like sometimes respiratory, sometimes gastrointestinal, and so on causing recurrent episodes of fever.

Digits of the hand establish a stronger influence on the dentoalveolar and orofacial structures than the digit of the feet, due to its proximity to the oral cavity. This explains why the toe sucking habit in our case did not cause severe malocclusion like presence of anterior open bites, posterior cross-bites, and so forth which are generally associated with finger sucking habits [13, 14]. However it caused deflection of the erupting permanent incisor and deformity of the great toe owing to the duration of the practiced habit.

The identification and intervention of this habit at the right time as in our case have helped in limiting the extent of malocclusion and also relieved the child from complaints of recurrent fever, which has restored the normal living in this child.

## 4. Conclusion

The aim of this paper is to report the influence of toe sucking habit on general health and dentofacial structures of the children.
